# The Association Between Cognitive Function and Oral Health in Home Dwellers and Nursing Home Residents: The HUNT Study

**DOI:** 10.1111/cdoe.13013

**Published:** 2024-10-14

**Authors:** Ernest Obeng Asante, Rannveig Sakshaug Eldholm, Marit Kolberg, Håvard Kjesbu Skjellegrind, Geir Selbæk, Xiao‐Mei Mai, Yue Chen, Yi‐Qian Sun

**Affiliations:** ^1^ Center for Oral Health Services and Research Mid‐Norway (TkMidt) Trondheim Norway; ^2^ Department of Clinical and Molecular Medicine, NTNU Norwegian University of Science and Technology Trondheim Norway; ^3^ Department of Neuromedicine and Movement Science, NTNU Norwegian University of Science and Technology Trondheim Norway; ^4^ Department of Geriatrics Clinic of Medicine, St. Olavs Hospital Trondheim Norway; ^5^ HUNT Research Centre, Department of Public Health and Nursing, NTNU Norwegian University of Science and Technology Levanger Norway; ^6^ Levanger Hospital, Nord‐Trøndelag Hospital Trust Levanger Norway; ^7^ Norwegian National Centre for Ageing and Health Vestfold Hospital Trust Tønsberg Norway; ^8^ Department of Geriatric Medicine Oslo University Hospital Oslo Norway; ^9^ Institute of Clinical Medicine, Faculty of Medicine University of Oslo Oslo Norway; ^10^ Department of Public Health and Nursing, NTNU Norwegian University of Science and Technology Trondheim Norway; ^11^ School of Epidemiology and Public Health, Faculty of Medicine University of Ottawa Ottawa Ontario Canada; ^12^ Department of Pathology Clinic of Laboratory Medicine, St. Olavs Hospital Trondheim Norway

**Keywords:** aged, cognitive dysfunction, dementia, neurocognitive disorders, oral health, population

## Abstract

**Objectives:**

To evaluate the relationships of cognitive function and care dependency with oral health in a Norwegian older adult population.

**Methods:**

This cross‐sectional study included 2623 participants aged 70 and older from the fourth wave of the Trøndelag health study (HUNT4 70+) and the city of Trondheim (Trondheim 70+). Neurocognitive disorders (NCDs) were diagnosed by clinical experts according to the DSM‐5 framework. Care dependency referred to nursing home residency. Oral health was assessed by using the Revised Oral Assessment Guide—Jönköping (ROAG‐J). Individuals were considered as ‘having oral problem’ if the score was two or three in at least one of the nine ROAG‐J items. Poisson regression was used to estimate prevalence ratios (PRs) and 95% confidence intervals (CIs).

**Results:**

The prevalence of having oral problems was 19% higher in participants with NCDs than those with normal cognitive function after adjusting for potential confounders (PR 1.19, 95% CI: 1.09–1.29). Further analysis showed a higher prevalence of having oral problems for home dwellers with NCDs (PR 1.23, 95% CI: 1.13–1.33) and nursing home residents (PR 1.32, 95% CI: 1.20–1.45) compared to home dwellers with normal cognitive function.

**Conclusions:**

NCDs were associated with an increased prevalence of oral problems in this Norwegian older adult population. The study suggests the need for increasing oral care for home dwellers with NCDs and nursing home residents.

## 1 Introduction

Oral health is a key component of overall well‐being. Good oral health promotes appropriate nutrition, supports clear speech, enhances self‐esteem and fosters positive social interactions [1, 2]. Orofacial pain and suffering resulting from poor oral health had a moderate impact on oral health‐related quality of life [3]. Good oral health also contributes to long‐term well‐being by reducing the risk of systemic health issues, as evidenced by reported links between periodontitis and cardiovascular diseases, diabetes and respiratory diseases in older adults [4]. Oral health becomes increasingly relevant with aging [5]. Thus, the aging population and the prevalent oral disease worldwide warrant investigation into the age‐related risk factors for poor oral health [6, 7].

Global trends indicate an increasing prevalence of neurocognitive disorders (NCDs), such as mild cognitive impairment (MCI) and dementia [8, 9]. In Norway, an estimated 100 000 cases of dementia were reported in 2020, with projections indicating a doubling of cases by 2050 [10]. Older adults with NCDs may be vulnerable to poor oral health due to reduced self‐care, medication side effects and dietary choices [11, 12, 13]. Accordingly, existing evidence seems to support the association between impaired cognitive function and adverse oral health [14], though conclusions from previous reviews were inconsistent [15, 16]. In addition, previous studies often fail to include dependent home‐dwelling individuals [17]. Dementia prevalence among nursing home residents in Norway was reported to be 84.3%, while the prevalence was 10.8% among individuals living at home [10]. The current Norwegian dementia strategy aims to support patients to live at home as long as possible, likely leading to a future rise in the prevalence of NCDs among home‐dwelling individuals [18]. These individuals are at risk of inadequate oral care [19].

Nursing home residents are clearly care‐dependent. Care dependency, in general, is also a suggested risk factor for poor oral health [20]. The high dementia prevalence in nursing homes of other developed countries has been associated with significant disability and dependence, leading to suboptimal oral self‐care and adverse oral conditions [13, 20, 21]. This care‐dependent population in Norway may also have high oral care needs [10, 22]. A previous Norwegian study found oral health status to be associated with the level of dependence and cognitive function in institutionalised older adults, albeit with a limited sample size [23].

Therefore, the aim of the study was to evaluate the association of cognitive function and care dependency with oral health in a Norwegian older adult population.

## 2 Methods

### 2.1 Study Population

The Trøndelag Health Study (HUNT), initiated between 1984 and 1986, is a large population‐based cohort study in Mid‐Norway [24]. The HUNT study collects data on demographics, self‐reported health, health‐related habits and biological measurements through questionnaires, interviews, clinical assessments and biological samples. The study has conducted four waves of data collection so far, with approximately 10 years between each follow‐up [24].

**FIGURE 1:**
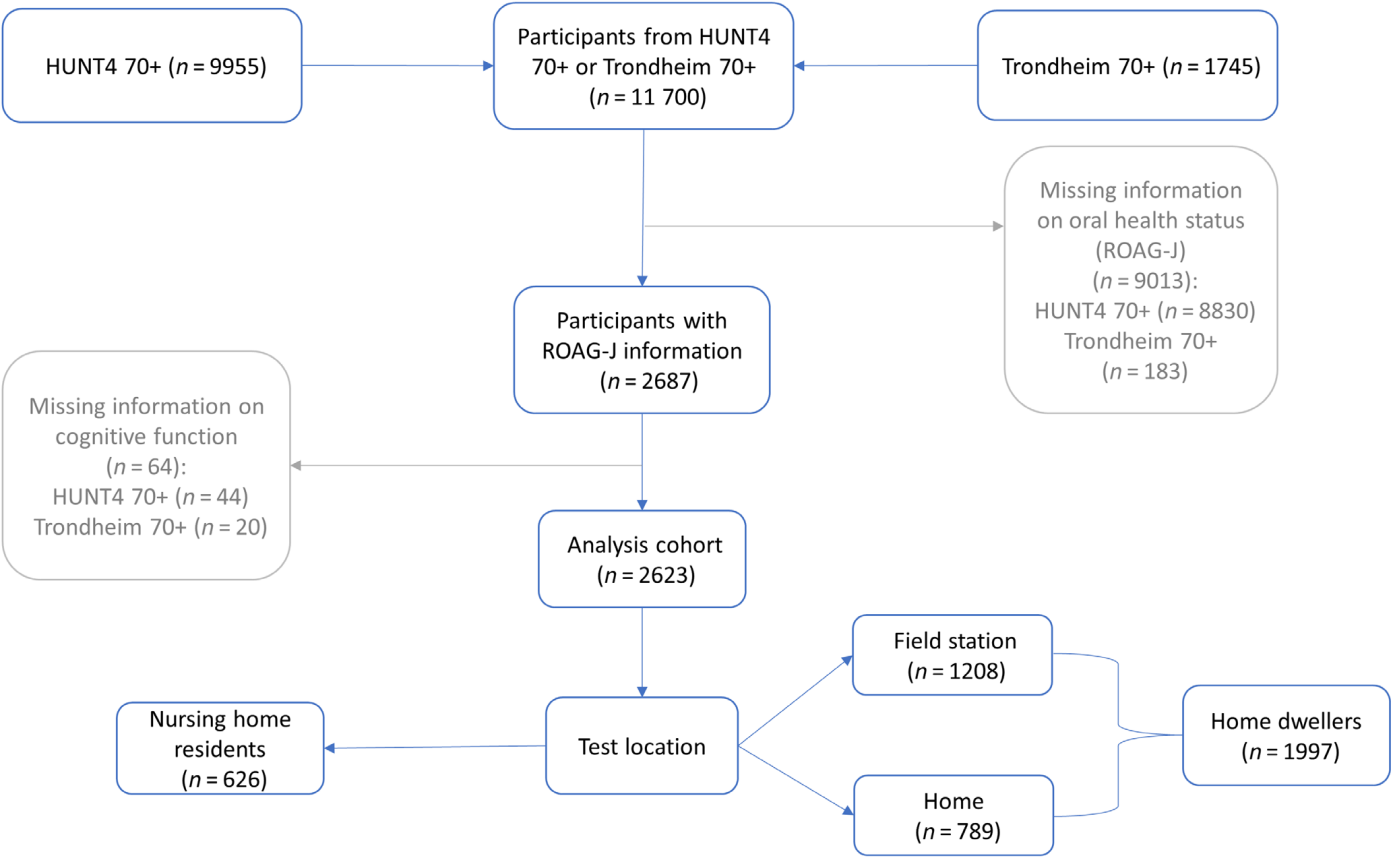
Flow chart of selection criteria for the analysis cohort of 2623 participants with information on cognitive function and oral health status (ROAG‐J). ROAG‐J, Revised Oral Assessment Guide—Jönköping.

During the fourth wave of the HUNT study (HUNT4; 2017–2019), inhabitants aged 70 years or older from the northern part of Trøndelag county (HUNT4 70+, *n* = 19 403) and the eastern district of Trondheim (Trondheim 70+, *n* = 5087), the largest city in the southern part of Trøndelag, were invited for additional assessments—including cognitive and physical function, oral health and nutritional status. The resulting population from both northern and southern Trøndelag generally represents Norway regarding demographics, socioeconomics and lifestyle [24]. Assessments were conducted at field stations set up in each municipality. Additionally, ambulatory teams were available to assess participants with difficulty coming to field stations at home or in nursing homes. In total, 11 700 (47.8% of all invited) participated in HUNT4 70+ (*n* = 9955) and Trondheim 70+ (*n* = 1745). For this study, 9013 participants with missing data on oral health status were excluded, followed by the exclusion of 64 participants with missing information on cognitive function. The final analysis cohort was comprised of 2623 individuals with complete information on cognitive function and oral health status. The substantial reduction of participants was because the oral health assessment was not performed at the field stations of HUNT4 70+. Of the 2623 participants, 1208 were examined at field stations, 789 at home and 626 at nursing homes. Home dwellers were defined as participants examined at the field station or home (*n* = 1997). A detailed selection process is outlined in Figure 1.

### 2.2 Cognitive Function Assessment

Clinical experts diagnosed MCI or dementia after a comprehensive clinical evaluation of participants' cognitive function. Participants were independently diagnosed by two clinical experts. If no consensus was reached, a third expert was consulted. The diagnosis was based on cognitive tests covering different domains and structured caregiver questionnaires according to the Diagnostic and Statistical Manual of Mental Disorders, Fifth Edition (DSM‐5) criteria [10]. In summary, cognitive function assessment covered cognition, daily life function, neuropsychiatric symptoms, subjective cognitive decline, symptom debut and course of the condition, and followed DSM‐5 criteria for diagnosing MCI (as mild neurocognitive disorder), dementia (as major neurocognitive disorder) and all the dementia subtypes [25]. The participants were classified into two main groups in the current study: those with normal cognitive function and those with NCDs comprising MCI and dementia. A detailed description of assessments and procedures for diagnosing NCDs in the participants can be found elsewhere [10].

### 2.3 Oral Health Assessment

The oral health status of participants was assessed using the Revised Oral Assessment Guide—Jönköping (ROAG‐J) [26, 27]. The ROAG‐J is a standardised screening tool developed for non‐dental healthcare professionals to facilitate oral health evaluation in older adults. The screening tool has demonstrated moderate to good validity and is clinically useful in screening oral health in older adults [26, 28]. Assessments were performed by non‐dental healthcare workers and nursing students after receiving comprehensive training from dentists and dental hygienists. The training program included tutoring sessions, practical exercises, instruction videos and manual. Both field station and ambulatory teams underwent identical training and had access to the same equipment. Oral examinations were conducted without a dental chair or specialised lighting in the field stations and ambulant visits. Participants sat in regular chairs and assessors utilised a flashlight along with the room's natural lighting. Notes were taken on the registration form following examination of each oral cavity area, referencing example descriptions and/or photos for each ROAG‐J item in the instruction manual.

The ROAG‐J screened nine items: lips, mucous membranes, tongue, saliva, voice, swallowing function, gums, teeth and dentures. The scoring of items was registered as 0–3. A score of 0 indicates that the observation of a specific item is irrelevant to the assessment; this was specified for voice, swallowing function, gums, teeth and dentures. For example, a score of 0 for gums, teeth or dentures represented the absence of teeth or dentures and, therefore, was not relevant to assess. A score of 1 is referred to as a healthy or normal condition. A score of 2 is considered moderate change/problem and a score of 3 is considered severe change/problem. In a healthcare setting, a score of 2 can be addressed by care personnel and a score of 3 indicates a need for referral to dental or medical professionals. More detailed information about the ROAG‐J can be found here [27]. For this study, ROAG‐J information was used to generate a binary outcome of ‘having oral problem’ (participants with a score of 2 or 3 for any of the nine items) or ‘no oral problem’ (the remaining participants). Additionally, to generate a continuous ROAG‐J outcome variable, scores 0–1 received 0 points, while scores of 2 and 3 were assigned 1 and 2 points respectively, resulting in a theoretical total point range of 0–18, used for supplementary analyses.

### 2.4 Covariates

Information on covariates was collected by questionnaires or at the clinical examination in HUNT4 70+ or Trondheim 70+. Sociodemographic factors included age (as a continuous variable), sex (female or male) and education (≤ 10, 11–13 and ≥ 14 years). Lifestyle factors included smoking status (never, former or current) and alcohol consumption (never, 1–4 times per month or ≥ 5 times per month). Trained personnel conducted height and weight measurements at the clinical examination. Height was measured in whole centimetres, and weight was measured to the nearest 0.1 kg. Body mass index (BMI) was calculated as weight divided by squared height, and participants were categorised as underweight or normal (< 25.0 kg/m^2^), overweight (25.0–29.9 kg/m^2^) or obese (≥ 30.0 kg/m^2^). Participants were also classified by test location (field station, participants' home or nursing home). Participants with missing data on these covariates were classified as an ‘unknown’ category for each variable and included in the statistical analyses. The categorisation of these covariates has been widely used in previous HUNT publications [29, 30].

### 2.5 Statistical Analyses

The characteristics of participants overall and stratified by home dwellers and nursing home residents were presented. The relationship between cognitive function and oral health status proxied by a binary ROAG‐J outcome (having oral problem vs. no oral problem) was evaluated using Poisson regression with robust error variance [31]. Crude and adjusted prevalence ratios (PRs) and 95% confidence intervals (CIs) were estimated in participants overall. In a further analysis, participants were classified into four groups based on cognitive function and care dependency. The four groups referred to home dwellers with normal cognitive function (reference group), home dwellers with NCDs, nursing home residents with normal cognitive function and nursing home residents with NCDs. The last two groups were merged as nursing home residents because there were only 15 nursing home residents with normal cognitive function.

A multivariable analysis was used to adjust for potential confounders, including age, sex, education, BMI, smoking status, alcohol consumption and test location (for overall analysis only). These covariates were considered potential confounders in the adjusted models. The selection of covariates was based on prior knowledge and an extensive review of the relevant literature [14, 15, 16]. Focus was placed on demographic factors and health‐related behaviours related to cognitive function and oral health in the framework of a directed acyclic graph. Supplementary analyses were performed using the total ROAG‐J point as a continuous outcome variable in negative binomial regression models, calculating ratios of means (RMs) with 95% CIs [32].

To address potential bias due to missing data on covariates (regarded as an ‘unknown’ category), multiple imputation by chained equations was used, assuming that data on covariates were missing at random. Ten imputed datasets were analysed, and the averaged estimates were presented [33].

Data were analysed using STATA/MP Version 17 (StataCorp LP, College Station, Texas).

## 3 Results

Table 1 shows the detailed characteristics of participants overall and stratified by home dwellers and nursing home residents. The mean age of all participants was 81.8 years, but it was higher among nursing home residents than home dwellers (87.3 vs. 80.0 years). Female representation was notably higher (61.6%). Home dwellers showed a higher proportion of overweight and had more years of education but fewer teetotallers than nursing home residents (after excluding unknowns). The prevalence of NCDs was high in the population (67.9%). Dementia was more common in participants from nursing homes than home dwellers (86.7% vs. 22.9%); the opposite was observed for MCI (10.9% vs. 35.8%).

**TABLE 1:** Characteristics of the analysis cohort overall and stratified by home dwellers and nursing home residents.

Characteristics	Total (*n* = 2623)	Home dwellers^a^ (*n* = 1997)	Nursing home residents (*n* = 626)
Age (years)	81.8 ± 8.0	80.0 ± 7.4	87.3 ± 7.1
Sex			
Female	1615 (61.6)	1180 (59.1)	435 (69.5)
Male	1008 (38.4)	817 (40.9)	191 (30.5)
Body mass index (kg/m^2^)			
Underweight or normal (< 25.0)	835 (31.8)	682 (34.2)	153 (24.4)
Overweight (25.0–29.9)	953 (36.3)	829 (41.5)	124 (19.8)
Obesity (≥ 30.0)	455 (17.3)	385 (19.3)	70 (11.2)
Unknown	380 (14.5)	101 (5.1)	279 (44.6)
Education (years)			
≤ 10	734 (28.0)	478 (23.9)	256 (40.9)
11–13	800 (30.5)	667 (33.4)	133 (21.2)
≥ 14	724 (27.6)	670 (33.6)	54 (8.6)
Unknown	365 (13.9)	182 (9.1)	183 (29.2)
Smoking status			
Never	792 (30.2)	664 (33.2)	128 (20.4)
Former	1023 (39.0)	861 (43.1)	162 (25.9)
Current	103 (3.9)	94 (4.7)	9 (1.4)
Unknown	705 (26.9)	378 (18.9)	327 (52.2)
Alcohol consumption			
Never	548 (20.9)	369 (18.5)	179 (28.6)
1–4 times per month	791 (30.2)	698 (35.0)	93 (14.9)
≥ 5 times per month	545 (20.8)	530 (26.5)	15 (2.4)
Unknown	739 (28.2)	400 (20.0)	339 (54.2)
Cognitive function			
Normal	841 (32.1)	826 (41.4)	15 (2.4)
Mild cognitive impairment	782 (29.8)	714 (35.8)	68 (10.9)
Dementia	1000 (38.1)	457 (22.9)	543 (86.7)

*Note:* Data are given as the number of participants (column percentage) or mean ± SD.

^a^ Home dwellers comprised individuals from field stations and their homes.

Table S1 shows the characteristics of all participants classified into four groups by home dwellers/nursing home residents and cognitive function. Overall, home dwellers with normal cognitive function were the youngest and had more years of education than those with NCDs or nursing home residents (after excluding unknowns).

The distributions of original ROAG‐J scores of nine items, overall and stratified by cognitive function, are illustrated in Figure S1. A higher prevalence of oral problems was observed across all ROAG‐J items in participants with NCDs compared to those with normal cognitive function. In summary, 65.2% of the study population had oral problems. The prevalence of having oral problems was higher in nursing home residents than in home dwellers (82.3% vs. 59.9%). The mean ROAG‐J point in all participants was 1.5 (range 0–11).

As shown in Table 2, 1294 out of 1782 (72.6%) participants with NCDs had oral problems compared with 417 out of 841 (49.6%) participants with normal cognitive function. The prevalence of having oral problems was 46% higher in participants with NCDs (PR 1.46, 95% CI: 1.36–1.58) than in those with normal cognitive function in the crude model. The association persisted in the adjusted model (PR 1.19, 95% CI: 1.09–1.29). Participants with MCI showed a 17% higher prevalence of having oral problems (adjusted PR 1.17, 95% CI: 1.07–1.27) and a 23% higher prevalence of having oral problems was found in participants with dementia (adjusted PR 1.23, 95% CI: 1.12–1.35). In the supplementary analysis (Table S2), NCDs were associated with a 20% increase in the mean ROAG‐J point (adjusted RM 1.20, 95% CI: 1.07–1.33).

**TABLE 2:** Association between cognitive function and oral health proxied by ROAG‐J as a binary outcome (*n* = 2623).

Cognitive function	*n*	Oral problem		Prevalence ratio (95% CI)	
		Cases	Prevalence (%)	Crude	Adjusted^b^
Normal	841	417	49.6	1.00 (Reference)	1.00 (Reference)
NCDs^a^	1782	1294	72.6	1.46 (1.36–1.58)	1.19 (1.09–1.29)
MCI	782	500	63.9	1.29 (1.18–1.41)	1.17 (1.07–1.27)
Dementia	1000	794	79.4	1.60 (1.49–1.73)	1.23 (1.12–1.35)

Abbreviations: 95% CI, 95% confidence interval; MCI, mild cognitive impairment; *n*, number of participants; NCDs, neurocognitive disorders; ROAG‐J, Revised Oral Assessment Guide‐Jönköping.

^a^ NCDs consisted of mild MCI and dementia.

^b^ Adjusted for age, sex, education, body mass index, smoking status, alcohol consumption and test location.

In the further analyses (Table 3), a higher prevalence of having oral problems was observed among home dwellers with NCDs (adjusted PR 1.23, 95% CI: 1.13–1.33) and nursing home residents (adjusted PR 1.32, 95% CI: 1.20–1.45) when compared to home dwellers with normal cognitive function. These associations persisted when using the total ROAG‐J point as the continuous outcome variable (Table S2).

**TABLE 3:** Oral health proxied by ROAG‐J as a binary outcome among participants classified by home dwellers/nursing home residents and cognitive function (*n* = 2623).

Groups	*n*	Oral problem		Prevalence ratio (95% CI)	
		Cases	Prevalence (%)	Crude	Adjusted^c^
Home dwellers with normal cognitive function	826	405	49.0	1.00 (Reference)	1.00 (Reference)
Home dwellers with NCDs^a^	1171	791	67.6	1.38 (1.27–1.49)	1.23 (1.13–1.33)
Nursing home residents^b^	626	515	82.3	1.68 (1.55–1.81)	1.32 (1.20–1.45)

Abbreviations: 95% CI, 95% confidence interval; MCI, mild cognitive impairment; *n*, number of participants; NCDs, neurocognitive disorders; ROAG‐J, Revised Oral Assessment Guide‐Jönköping.

^a^ NCDs consisted of MCI and dementia.

^b^ Nursing home residents having normal cognitive function (*n* = 15) or NCDs (*n* = 611).

^c^ Adjusted for age, sex, education, body mass index, smoking status and alcohol consumption.

The imputed data analysis for missing values on covariates yielded similar results to the main results (Table S3 vs. Table 2), with a slight increase in the prevalence of having oral problems for nursing home residents (adjusted PR 1.40, 95% CI: 1.28–1.53) (Table S3 vs. Table 3).

## 4 Discussion

In this cross‐sectional study, NCDs were associated with poorer oral health status in a Norwegian older adult population aged ≥ 70 years. Further analyses showed a higher prevalence of oral health problems in home dwellers with NCDs and nursing home residents compared to home dwellers with normal cognitive function.

Consistent with the current findings, cognitive decline has been independently associated with periodontal disease, dental caries, tooth loss and oral cavity discomfort in other cross‐sectional studies [34, 35, 36]. In longitudinal studies, poor cognitive function predicted poor self‐rated oral health and subsequent tooth loss [37, 38]. Similarly, dementia was associated with a higher risk of periodontitis during a 10‐year follow‐up [39], further supported by a meta‐analysis confirming the association between cognitive decline and periodontal disease severity [40]. Few studies have used non‐dental oral assessment tools to appraise the relationship between cognitive function and oral health. Notably, no association was found between cognitive decline and oral health assessed using the oral health assessment tool (OHAT) in a 4‐year follow‐up, which might be because only a single cognitive domain (memory) had been assessed [41].

The result of the further analyses among participants classified by cognitive function and care dependency (nursing home residents vs. home dwellers with normal cognitive function) is consistent with the frequently reported poor oral health among care‐dependent older adults [17, 21]. The current study could not explicitly distinguish home dwellers receiving home care from independent home dwellers, yet those with NCDs exhibited poor oral health. The poor oral health of home dwellers with NCDs can be attributed to a significantly decreased utilisation of dental services and deterioration in oral hygiene [42, 43]. Moreover, formal care, such as professional home care services, may be inadequate or deficient for home dwellers with NCDs [19]. As a result, newly admitted nursing home residents often have compromised oral health status, which also progresses to severe oral problems within several months after admission [44]. The deterioration of oral health in nursing home residents has been associated with barriers to providing oral care. Frequent barriers to sufficient oral care, such as resistance from the residents, care providers' lack of knowledge of providing oral care and overburdened staff, have been outlined in a meta‐analysis by Hoben et al. [45] Therefore, consistent with recent systematic reviews [17, 21], oral care needs are likely required for individuals with care dependency such as both home dwellers with NCDs and nursing home residents. The lack of oral care likely led to the observed increased prevalence of having oral problems in these groups [19, 22]. The slightly better oral health status among home dwellers with NCDs than nursing home residents in the current and other studies may be due to the less severity of NCDs or less frailty among home dwellers [46, 47].

Cognitive function may influence oral health through a combination of factors, mainly its impact on self‐care, including oral care. The current study suggests that there is a strong association between NCDs and dependency related to functional disability in older adults, with 86.7% of nursing home residents having dementia. This dependency is significantly associated with a decline in oral hygiene and an increased prevalence of oral diseases in older adults [13]. Additionally, drugs used to manage dementia symptoms (antipsychotics, antidepressants, anxiolytics and anticonvulsants) have been associated with a partial absence of saliva [48]. Gil‐Montoya et al. [11] found an association between NCDs and drug‐induced xerostomia (subjective dry mouth sensation) and clinical signs of dry mouth, which negatively impacts oral health [49]. NCDs have also been associated with changes in dietary habits and patterns, with a documented preference for sweets and carbohydrates that further deteriorate oral health [12, 50].

The study has the following strengths. A broad population of older adults was studied. The prevalence of dementia among the nursing home residents in this HUNT Study (86.7%) was similar to the reported prevalence among nursing home residents for the Norwegian population (84.3%) [10]. The use of ambulatory teams during data collection for individuals with difficulty getting to field stations prevented the loss of care‐dependent or sick home dwellers and nursing home residents. Thus, this study provides insights into the relationship between cognitive function and oral health in both home‐dwelling and institutionalised older adults. Also, clinical experts assessed participants' cognitive function and NCDs were diagnosed according to the DSM‐5 framework, ensuring a comprehensive and standardised assessment. Additionally, oral health was evaluated using ROAG‐J, a validated oral assessment tool in population studies [26, 28].

However, some limitations also need to be addressed. First, there was a potential selection bias with relatively ‘healthy individuals’ being underrepresented. Most participants excluded from the study were from the field stations (95%), particularly in HUNT4 70+, due to a lack of ROAG‐J information. Data showed that the excluded participants were younger, had a smaller sex disparity and consumed more alcohol than the analysis cohort (after excluding unknowns) (Table S4). Second, while the ROAG‐J has demonstrated good validity [26, 28], it is crucial to acknowledge the limitations as further research on validity has been recommended for all existing non‐dental oral health assessment tools [51]. Specifically, there was potential variability in the assessment of ROAG‐J items such as teeth/dentures and tongue, with less consistent agreement observed between personnel [26, 28]. Third, there was missing information on some adjusted covariates and unmeasured confounding, such as a complete comorbidity profile of the participants. However, analyses using multiple imputation of missing values of covariates by chained equations provided similar results (Table S3). Fourth, exact information on care dependency among home dwellers was lacking, making the use of NCDs as a proxy. Finally, causality could not be determined due to the cross‐sectional study design.

In summary, the study showed that older adults with NCDs had a poorer oral health status than those with normal cognitive function in this Norwegian population. It also showed that oral problems were most prevalent among the care‐dependent older adults. Policymakers may consider increasing oral care for home‐dwelling older adults with NCDs and nursing home residents.

## Author Contributions

E.O.A., X.‐M.M. and Y.‐Q.S. performed the literature search and contributed to the study design. M.K., H.K.S., G.S., X.‐M.M. and Y.‐Q.S. were responsible for data collection. E.O.A. and Y.‐Q.S. conducted statistical analyses, interpreted results and wrote the initial draft of the manuscript. E.O.A., R.S.E., M.K., H.K.S., G.S., X.‐M.M., Y.C. and Y.‐Q.S. participated in the data interpretation and the manuscript writing with important intellectual content and approved the final version.

## Ethics Statement

The study was approved by the Norwegian Regional Committees for Medical and Health Research Ethics (no. 31812). All HUNT participants have signed informed consent for participation and the use of data in research.

## Conflicts of Interest

The authors declare no conflicts of interest.

## Supporting information


Appendix S1.

## Data Availability

Data from the HUNT Study used in research projects will, when reasonably requested by others, be made available on request to the HUNT Data Access Committee. The HUNT data access information describes the policy regarding data availability (https://www.ntnu.edu/hunt/data).
