# Supplementary material for: The Association Between Cognitive Function and Oral Health in Home Dwellers and Nursing Home Residents: The HUNT Study
Source: Community Dent Oral Epidemiol. 2024 Oct 14;53(1):98–105. doi: 10.1111/cdoe.13013 (PMC11754152; doi:10.1111/cdoe.13013)
Supplement: Supplementary file 1 — Appendix S1. [file CDOE-53-98-s001.pdf]

Supplementary table 1. Characteristics of participants categorized by home dwellers/nursing home residents and cognitive function (n=2623)

Characteristics	Home dwellers		Nursing home residents	
	Normal cognitive function (n=826)	NCDs[a] (n=1171)	Normal cognitive function (n=15)	NCDs[a] (n=611)
Age (years)	77.3 ± 6.0	82.0 ± 7.7	90.4 ± 4.0	87.2 ± 7.2
Sex				
Female	464 (56.2)	716 (61.1)	12 (80.0)	423 (69.2)
Male	362 (43.8)	455 (38.9)	3 (20.0)	188 (30.8)
Body mass index (kg/m^2)				
Underweight or normal (<25.0)	280 (33.9)	402 (34.3)	6 (40.0)	147 (24.1)
Overweight (25.0–29.9)	379 (45.9)	450 (38.4)	3 (20.0)	121 (19.8)
Obesity (≥30.0)	151 (18.3)	234 (20.0)	2 (13.3)	68 (11.1)
Unknown	16 (1.9)	85 (7.3)	4 (26.7)	275 (45.0)
Education (years)				
≤10	100 (12.1)	378 (32.3)	5 (33.3)	251 (41.1)
11–13	299 (36.2)	368 (31.4)	3 (20.0)	130 (21.3)
≥14	383 (46.4)	287 (24.5)	2 (13.3)	52 (8.5)
Unknown	44 (5.3)	138 (11.8)	5 (33.3)	178 (29.1)
Smoking status				
Never	304 (36.8)	360 (30.7)	5 (33.3)	123 (20.1)
Former	403 (48.8)	458 (39.1)	5 (33.3)	157 (25.7)
Current	41 (5.0)	53 (4.5)	0 (0.0)	9 (1.5)
Unknown	78 (9.4)	300 (25.6)	5 (33.3)	322 (52.7)
Alcohol consumption				
Never	87 (10.5)	282 (24.1)	4 (26.7)	175 (28.6)
1–4 times per month	325 (39.3)	373 (31.9)	5 (33.3)	88 (14.4)
≥5 times per month	323 (39.1)	207 (17.7)	1 (6.7)	14 (2.3)

Unknown	91 (11.0)	309 (26.4)	5 (33.3)	334 (54.7)

Data are given as the number of participants (column percentage) or mean ± SD.

NCDs, neurocognitive disorders

[a]NCDs consisted of mild cognitive impairment and dementia.

Supplementary table 2. The relationship between cognitive function and oral health proxied by total ROAG-J point as a continuous variable (n=2623)

Sample	Cognitive function	n	Total ROAG-J point	Ratio of means (95% CI)	
			Mean (range)	Crude	Adjusted[c]
**Total**	Normal	841	1.0 (0 - 8)	1.00 (Reference)	1.00 (Reference)
	NCDs[a]	1782	1.8 (0 – 11)	1.79 (1.63 – 1.98)	1.20 (1.07 – 1.33)
	MCI	782	1.4 (0 – 10)	1.38 (1.23 – 1.54)	1.14 (1.01 – 1.27)
	Dementia	1000	2.1 (0 – 11)	2.12 (1.91 – 2.35)	1.32 (1.16 – 1.51)
**Subgroups**					Adjusted[d]
Home dwellers wth normal cognitive function		826	1.0 (0 – 8)	1.00 (Reference)	1.00 (Reference)
Home dwellers with NCDs[a]		1171	1.6 (0 – 10)	1.61 (1.45 – 1.78)	1.28 (1.15 – 1.43)
Nursing home residents[b]		626	2.1 (0 – 11)	2.21 (1.97 – 2.47)	1.33 (1.16 – 1.53)

95% CI, 95% confidence interval; MCI, mild cognitive impairment; n, number of participants; NCDs, neurocognitive disorders; ROAG-J, Revised Oral Assessment Guide-Jönköping

[a]NCDs consisted of MCI and dementia.

[b]Nursing home residents having normal cognitive function (n=15) or NCDs (n=611)

[c]Adjusted for age, sex, education, body mass index, smoking status, alcohol consumption and test location.

[d]Adjusted for age, sex, education, body mass index, smoking status and alcohol consumption.

Supplementary table 3. The relationship between cognitive function and oral health proxied by ROAG-J as a binary outcome.

Analyses after performing multiple imputation of missing data in covariates.

Sample	Cognitive function	n	cases	Prevalence (%)	Prevalence (95% CI) Crude	Adjusted[c]
**Total**	Normal	841	417	49.6	1.00 (Reference)	1.00 (Reference)
	NCDs[a]	1782	1294	72.6	1.46 (1.36 – 1.58)	1.19 (1.09 – 1.29)
	MCI	782	500	63.9	1.29 (1.18 – 1.41)	1.17 (1.07 – 1.28)
	Dementia	1000	794	79.4	1.60 (1.49 – 1.73)	1.23 (1.13 – 1.35)
**Subgroups**						Adjusted[d]
Home dwellers with normal cognitive function		826	405	49.0	1.00 (Reference)	1.00 (Reference)
Home dwellers with NCDs[a]		1171	791	67.6	1.38 (1.27 – 1.49)	1.24 (1.14 – 1.34)
Nursing home residents		626	515	82.3	1.68 (1.55 – 1.81)	1.40 (1.28 – 1.53)

95% CI, 95% confidence interval; MCI, mild cognitive impairment; n, number of participants; NCDs, neurocognitive disorders; ROAG-J, Revised Oral Assessment Guide-Jönköping

[a]NCDs consisted of MCI and dementia.

[b]Nursing home residents having normal cognitive function (n=15) or NCDs (n=611)

[c]Adjusted for age, sex, education, body mass index, smoking status, alcohol consumption and test location.

[d]Adjusted for age, sex, education, body mass index, smoking status and alcohol consumption.

Supplementary table 4. Characteristics of participants excluded from the study compared with the analysis cohort.

Characteristics	Excluded from the study (n=9077)	Analysis cohort (n=2623)
Age (years)	77.0 ± 5.7	81.8 ± 8.0
Sex		
Female	4813 (53.0)	1615 (61.6)
Male	4264 (47.0)	1008 (38.4)
Body mass index (kg/m^2)		
Underweight or normal (<25.0)	2743 (30.2)	835 (31.8)
Overweight (25.0–29.9)	4038 (44.5)	953 (36.3)
Obesity (≥30.0)	2001 (22.0)	455 (17.3)
Unknown	295 (3.2)	380 (14.5)
Education (years)		
≤10	2573 (28.3)	734 (28.0)
11–13	4038 (44.5)	800 (30.5)
≥14	2349 (25.9)	724 (27.6)
Unknown	117 (1.3)	365 (13.9)
Smoking status		
Never	3337 (36.8)	792 (30.2)
Former	4819 (53.1)	1023 (39.0)
Current	581 (6.4)	103 (3.9)
Unknown	340 (3.7)	705 (26.9)
Alcohol consumption		
Never	1787 (19.7)	548 (20.9)
1–4 times per month	5022 (55.3)	791 (30.2)
≥5 times per month	1763 (19.4)	545 (20.8)
Unknown	505 (5.6)	739 (28.2)
Test location		
Field station	8604 (95.0)	1208 (46.1)
Home	185 (2.0)	789 (23.9)
Nursing home	266 (3.0)	626 (30.1)

Data are given as the number of participants (column percentage) or mean ± SD.

Supplementary figure 1. ROAG-J information of participants in the analysis cohort

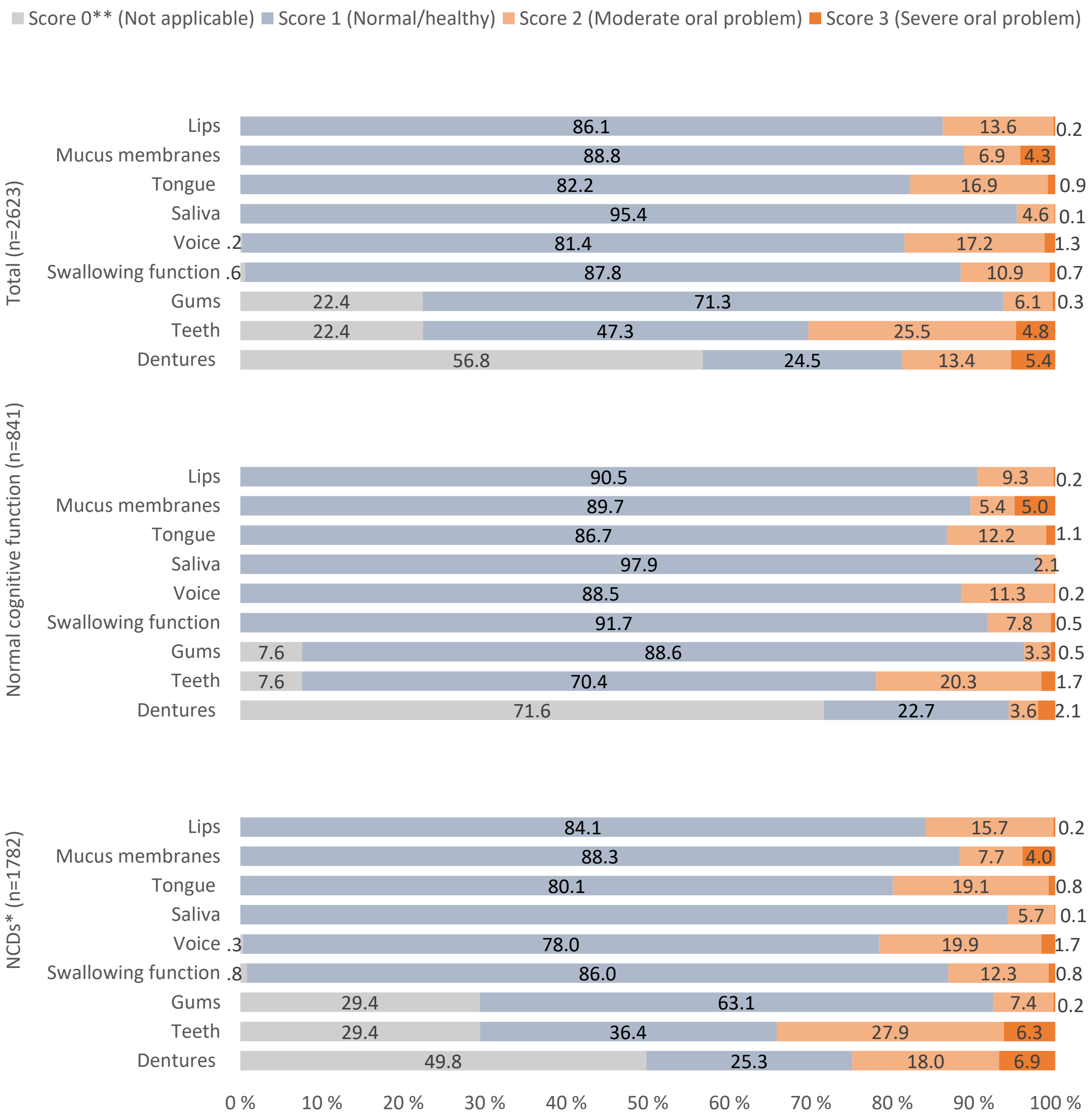

Data for ROAG-J items are given as the row percentage.

*NCDs consisted of MCI and dementia.

**A score of 0 is given if the observation of a ROAG-J variable is not relevant and/or not assessed; this applies explicitly to the assessment of voice, swallowing function, gums, teeth and dentures.

Original ROAG-J score for the sum of all 9 items: Total sample (mean 9.5 ± standard deviation 1.8); normal cognitive function (9.1 ± 1.4); NCDs (9.7 ± 2.0).
